# The impact of living alone on older adults’ mental health and the mediating role of healthy diet

**DOI:** 10.3389/fpsyt.2025.1562487

**Published:** 2025-05-29

**Authors:** Jichao Zheng, Zeqiang Ni

**Affiliations:** ^1^ Department of Economics Research, Anhui Academy of Social Sciences, Hefei, Anhui, China; ^2^ School of Economics and Management, Hefei University, Hefei, Anhui, China

**Keywords:** living alone, mental health, depressive symptoms, healthy diet, older adults

## Abstract

**Background:**

More than 30 million older people in China live alone. Research has shown that living alone can lead to poor mental health and that there are mediating variables, such as healthy diet, between living alone and mental health. Therefore, it is important to examine the role of these mediating variables between living alone and mental health to help us develop cost-effective mental health interventions.

**Methods:**

We used the multiple regression method in the R software to test the effect of living arrangements on depression by placing the living arrangements variable, the covariates, and the healthy diet variables into the regression equation. We then used the structural equation method with the R software package lavaan to derive the path coefficients of living arrangements on depression scores through the three mediating paths of fruits, vegetables, and nuts consumption. We used bootstrapping to derive confidence intervals for the coefficients.

**Results:**

Multiple regression results showed that the coefficient of the effect of living alone on depressive symptoms was 1.02. At the same time, all three variables of a healthy diet can alleviate depression, i.e., consuming more fruits, vegetables, and nuts helped to reduce depression scores among older adults. In the mediation analysis, living alone affects the mental health of older adults through three channels, namely, fruit consumption, vegetable consumption, and nuts consumption, respectively, and the indirect effects of these three channels accounted for 15.47% of the total impact of living alone on depression scores of older adults, with vegetable intake having the most significant effect on depression scores, accounting for 8.35% of the total impact, followed by nuts intake and fruits intake.

**Conclusion:**

Older people living alone are a vulnerable group with poor mental health and require a variety of interventions to improve their mental health. Healthy eating is one possible avenue of intervention; the Government should take diversified initiatives to enhance the healthy diet of older persons living alone.

## 1 Background

Globally, it is increasingly common for older persons to live alone. For example, in 2010, Europe had the highest proportion of older people living alone (28%), followed by North America (25%) (1). By 2022, the number of people over 65 in China has reached 209.78 million, accounting for 14.9% of the total population (2). In the past, in accordance with filial tradition, Chinese older adults generally lived with their children (3). However, as a result of declining fertility rates, older persons have fewer adult children than ever before (4). Meanwhile, a large number of young people are moving into the cities (5). It is estimated that 260 million people have moved from the countryside to the cities (6). As a result, more and more Chinese older adults are living alone (7). According to the seventh population census in 2020, there were 29.94 million households of older persons living alone in the country, compared to 14.44 million ten years ago (8).

Mental disorders are a major contributor to the global health-related burden, with depression and anxiety disorders being significant contributors to this burden (9). Depression affects health and quality of life (10). Depression is characterized by low mood, low energy, sadness, and inability to enjoy life (11). In terms of years lived with disability(YLD), depressive disorders are one of the three leading causes of YLD (12) in 2017. Aging-related processes increase the likelihood of depression (13). As a result, depression is widespread among older adults (14).The prevalence of depression is also high among older people living alone. A survey based on older people in the Shanghai community showed that the prevalence of depression among older people living alone was 26.9% (15). So it is very important to study the mental health of older people living alone. Some academic studies have been conducted on the mental health of older people living alone. Some scholars have focused on social support and social network support for older adults living alone (16). Other researchers have focused on caring for older adults living alone (17).

At the same time, there are mediating variables between living alone and mental health, which some scholars have explored. These variables include physical activity, social participation, intergenerational support, and social activities (18, 19). Other scholars have focused on the mediating role of sleep quality and anxiety in the relationship between living alone and depression (20). Diet plays a very important role in people’s lives, and some studies have shown that certain plant components in the diet are protective against depression (21). For example, some scholars believe that a high intake of fruits and vegetables will reduce depression risk (22). Others believe that the Mediterranean diet enhances mental health and reduces depression (23). Surprisingly, however, few studies have focused on the mediating role of healthy eating between living alone and depression, and this paper will fill a research gap in this area.

## 2 Literature review and hypotheses

### 2.1 Direct effect of living alone on depressive symptoms

There are several pathways in which living alone affects mental health. First, living alone and loneliness are two sides of the same coin; living alone is an objective and structural indicator of social isolation, while loneliness is a subjective perception of social isolation (24). One study found one of the most predictive factors of loneliness was living alone (25). And loneliness is a risk factor for depressive symptoms (26). Thus, in general, we can assume that living alone was associated with depression (27). Empirical research also supports the above conclusion, with one study finding a significant increase in depressive symptoms in both men and women after starting to live alone (28).

Second, living alone reduces social interactions and family support for older adults, and studies have shown that men who lack stronger family and friend social networks have a high risk of depression (29). Since most older people living alone are widowed (30)., they have lost the most important social relationship in their later life, i.e., the spousal relationship, which affects their social interaction behavior. For example, when a spouse dies, survivors can find it difficult to maintain previous social relationships (31). Therefore, living alone generally reduces social contact (32). In addition, people who live alone tend to receive less family support than those living with their families (33). Meanwhile, people living alone also lack practical or emotional help from others (34).

As mentioned above, living alone can lead to loneliness and reduced social interactions and family support. As a result, older adults who live alone show more depressive symptoms than those who live with others (35). A study in China suggests that living alone is associated with a higher risk of depressive symptoms, especially among people with less financial support (36). Based on the above literature review, we formulate the following hypotheses:

H1: Living alone can lead to depression in older adults.

### 2.2 The impact of living alone on healthy diet

There are various concepts of a healthy diet. Some studies suggest that a healthy diet is based on plant foods and is characterized by a high intake of vegetables and fruits (37). Some scholars also consider the Mediterranean diet to be healthy (38). The Mediterranean diet is characterized by plant-based foods, including fruits, vegetables, nuts, and olive oil (39). In fact, the traditional Chinese diet is also plant-based (40). A meta-analysis found that a healthy Chinese diet consisting of a plant-based diet reduced the risk of adverse health outcomes (41).The Chinese Dietary Guidelines recommend eating plenty of vegetables and fruits, as well as eating nuts regularly (42). Overall, we can assume that in the Chinese context, a healthy diet means consuming more vegetables, fruits, and eating nuts regularly.

Living alone affects a healthy diet for several reasons: first, older adults living alone have lower incomes than those living with spouses (43). Some studies have found that people who live alone are more likely to be female and have lower education and income levels (44). A study in China also found that people living alone had lower social status and income (45). On the other hand, healthy foods are usually more expensive (46). One study found that the average price per serving of healthy foods (vegetables, fruits, etc.) is almost double that of unhealthy foods (47). Therefore, older adults living alone are at risk of not having adequate access to healthy foods such as vegetables, fruits, and nuts due to their lower incomes.

Second, psychological and cultural factors associated with living alone may also influence the consumption of healthy foods. Residential patterns can affect one’s daily living patterns (48). From a psychological perspective, people may feel it is not worth cooking for only one person (49). Also, because people who live alone lack the motivation to cook, they tend to eat a smaller variety of foods (50). From a gender-cultural analysis perspective, in cases where women take on the task of cooking, when women are widowed and live alone, they are often not used to eating alone and may not see the purpose of cooking (51). For some men, cooking while living alone can be a “chore” or “hassle,” and some choose to use frozen meals, which can affect the quality of their diet (52). Moreover, fruits and vegetables cannot be preserved for a long time and need to be purchased outside frequently. And some studies have found that older people living alone tend to go out less often (53). As a result, it may be difficult for older adults to buy fresh ingredients.

It is for the above reasons that many studies have found that people who live alone have lower intake of fruits, vegetables, etc. (54). One study found that people who lived alone were the least likely to consume adequate amounts of vegetables and fruits (55). Based on the above literature review, we propose the following hypotheses:

H2a1: Living alone reduces fruit consumption.
H3a2: Living alone reduces vegetable consumption.
H4a3: Living alone reduces the consumption of nuts.

### 2.3 The role of healthy diet on depressive symptoms

The role of a healthy diet in mental health is manifested at two levels. First, consuming specific types of food, such as vegetables and fruits, helps to reduce mental stress and maintain mental health (56). In contrast, inadequate intake of fruits and vegetables can lead to poor mental health (57). Second, the greater the variety of foods, the lower the level of depression (58).

A healthy diet affects mental health primarily through anti-inflammatory and antioxidant mechanisms. Inflammation and oxidative stress are thought to be potential biological pathways associated with depression (59). Inflammation increases susceptibility to depression (60). And it is argued that inflammation and depression are thought to fuel each other (61). Meanwhile, oxidative stress is one of the major causes of depression (62). And antioxidants are an important physiological indicator of depression (63).

A healthy diet reduces depression and improves mental health through physiological mechanisms such as anti-inflammatory and antioxidant effects. First, a healthy diet has antioxidant effects; for example, plant-based foods are rich in antioxidants (64). Numerous studies have shown that the flavonoid content of fruits and vegetables helps reduce oxidative cell damage (65). Secondly, healthy foods have anti-inflammatory effects. A healthy diet helps maintain lower concentrations of inflammatory mediators, and a higher intake of vegetables, fruits, nuts, and fish is associated with lower inflammation (66). One study suggests that a year-long Mediterranean diet reduces inflammation and has a systematically beneficial effect on the health status of older adults (67).

At the same time, specific components of plant-based diets benefit mental health, such as plant polyphenols that have a protective effect against depression (68). Dietary fiber also produces short-chain fatty acids that have anti-inflammatory and anti-aging effects (69). Therefore, it has been suggested that high consumption of vegetables, fruits, and fish is protective against risk factors for developing CES-D depression (70). It has also been found that healthy eating patterns, such as the Mediterranean diet, can reduce the risk of depression (71). Overall, it has been suggested that diet plays an important role in the prevention and treatment of depression (72). Based on the above literature review, we formulate the following hypotheses:

H2b1: Consumption of fruits may reduce depression.
H3b2: Consumption of vegetables may reduce depression.
H4b3: Consumption of nuts may reduce depression.

## 3 Materials and methods

### 3.1 Data sources

This study used data from the China Longitudinal Healthy Longevity Survey (CLHLS) survey (2017–2018). The CLHLS is a tracking survey of older adults organized by Peking University, which is available to scholars for research purposes (73). In the most recent follow-up survey (2017–2018), 15,874 people were interviewed. The questionnaire provided information on family structure, living arrangements, activities of daily living (ADLs), self-rated health, care needs and costs, socialization, diet, drinking behaviors, psychosocial characteristics, financial resources, and other information. China’s Law on the Protection of the Rights and Interests of Older Adults defines older adults as those over 60. Therefore, according to our study objectives, we limited our sample to older adults over 60. At the same time, since we are studying the dietary structure of older people, which is strongly influenced by dental status, and to avoid dental problems confounding the relationship between the variables, we restricted the sample to older adults with 20 natural teeth or wearing dentures. 20 was chosen because studies have concluded that maintaining ≥ 20 teeth is important for mastication function (74), and Japan has even launched the 8020 campaign, an initiative to retain 20 natural teeth at age 80 (75).

### 3.2 Dependent variables

In the CLHLS survey, the depressive symptoms variable was measured using a simplified version of the CES-D scale with ten questions. The scale is designed to measure depressive symptoms in the general population (76). The 10-item CES-D scale showed good predictive accuracy compared to the full version of the CES-D, which has 20 items (77). The scale consists of seven negatively scored questions and three positively scored questions. The negative-scoring questions include questions such as “Are you bothered by things that don’t usually bother you?”; the positive-scoring questions include questions such as “Are you hopeful about the future? “ Five options were provided for each question: always, often, sometimes, rarely, and never. We assigned each option a value of 1, 2, 3, 4, or 5 for the positive-scoring questions and 5, 4, 3, 2, or 1 for the negative-scoring questions. We summarized the answers to these ten questions and constructed a continuous variable ranging from 10 to 50, with larger values indicating more significant levels of depression. The ten-item depression scale had an Alpha value of 0.8, indicating that the data had sufficient reliability to be analyzed.

### 3.3 Explanatory variables

The first explanatory variable is living alone, and the questionnaire includes the question, “ Who do you currently live with? “. The options are: 1. family (including live-in nannies); 2. living alone; and 3. nursing home. We defined respondents who chose 2 as living alone and assigned a value of 1, whereas respondents who chose 1 and 3 were defined as not living alone and assigned a value of 0.

Next are the fruit and vegetable consumption variables; the questionnaire contains the question “Do you eat fresh fruits and vegetables regularly?”. The options are: 1. daily/almost daily; 2. Often; 3. Occasionally; 4. seldom or never. We assign values of 4-1, respectively.

Finally, we obtained the nuts variable. The questionnaire included “How often do you eat nuts?”. Nuts here include peanuts/walnuts/chestnuts/watermelon seeds, etc. The options for this question were: 1. almost every day; 2. not every day but at least once a week; 3. not every week but at least once a month; 4. not every month but sometimes; and 5. rarely or never. We assign values of 5-1, respectively.

CLHLS is a well-established database and its dietary data are used in many studies. For example, one study found that increased intake of vegetables and fruits improved cognitive function in Chinese oldest adults (78). Another study, based on CLHLS data, found that maintaining high food variety reduced the incidence of frailty in older Chinese adults (79).

### 3.4 Covariates

As shown in **Table 1**, we categorize covariates into three main groups. The first group is demographic background factors, including gender, age, and marital status. The second group is the respondent’s socio-economic status, measured by several indications such as years of schooling, residence, perceived income adequacy, and visits (80). The third dimension is the respondent’s health status, which mainly includes self-rated health, whether they drink alcohol or not, and instrumental activities of daily living.

**Table 1:** Summary of the covariates.

Variables	Original question	Code
Gender	Respondent’s gender.	Male=1, Female=0.
Age	Current Age.	The variable takes the value of actual age.
Marital status	Current marital status.	Married and living with spouse =1, Others=0.
Years of schooling	How many years did you attend school?	The variable takes the value of the respondent’s years in school.
Residence	Type of the respondent’s current place of residence:1.city;2.town;3. rural.	Rural=1, others=0.
Perceived income adequacy	Is all your financial support sufficient to cover your daily expenses?	Yes=1, No=0.
Visits	Do your children visit you often?	The variable takes the value of 1 if one child regularly visits the respondent, 2 if there are two children who regularly visit the respondent, and so on.
Self-rated health	How do you rate your health at present?	The answers are very good, good, so so, bad, and very bad; each answer was coded as 5, 4, 3, 2, or 1.
Drink	Do you drink alcohol at the present?	Yes=1, No=0.
Iadl	Can you visit your neighbor’s house, go shopping, cook, do laundry, walk 1000 meters in a row, lift something weighing about 5 kilograms, squat down and stand up three times in a row, and travel by public transportation all by yourself? There are eight questions.	The answers to these questions were: yes, independently; yes, but need some help; and can’t do it. The above options are assigned values 0-2, respectively. We summed the answers to the eight questions to obtain a score of 0-16; the higher the score, the worse the respondent’s ability to perform daily activities.

### 3.5 Research methodology

First, we visualized the effect of living alone on depressive symptoms by describing the data on various variables for the overall sample, the sample of older adults living alone, and the sample of older adults not living alone.

**Figure 1:**
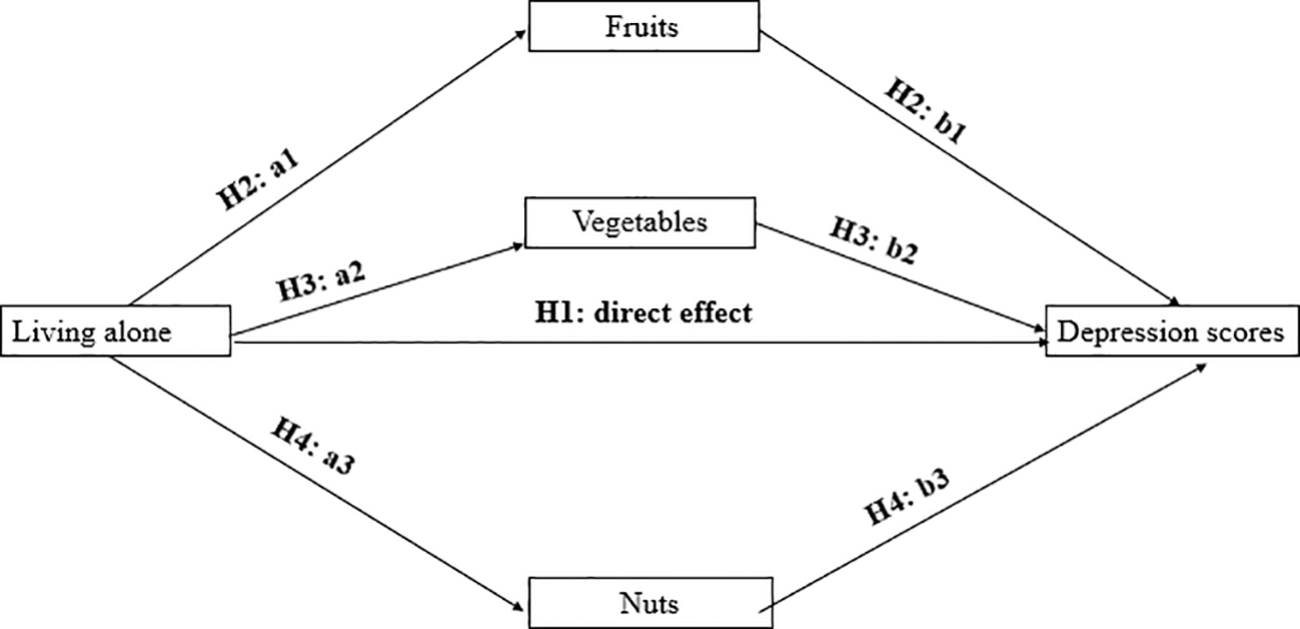
Empirical research hypothesis.

Second, we tested the effect of the living alone variable on depressive symptoms by performing a multiple regression using the R software. As shown in **Figure 1**, we included the living alone variable and the covariates in the regression equation. Then, we placed the dietary variables such as fruits, vegetables, and nuts into the regression equation. In this way, we tested the H1 hypothesis by regression analysis.

Finally, as hypothesized in the previous section, we believe that living alone indirectly affects depressive symptoms through three mediating pathways, including fruit, vegetable, and nut consumption. We will use the structural equation approach in the R package lavaan to derive the pathway coefficients of living alone on depression and the bootstrap approach to derive confidence intervals for the coefficients. Thus, through mediated effects analysis, we test hypotheses H2, H3, and H4, respectively.

## 4 Results

### 4.1 Data description

After excluding samples with missing values on the variables included in the analysis, the samples used in our study had 6,162 respondents, of which 9,90 lived alone and 5,172 lived in nursing homes or with their families.

The depression levels of the different groups are shown in **Table 2** below. The highest level of depression is found among older adults living alone, with an average of 22.83, while the lowest level of depression is found among those living with their families or in nursing homes, with an average of 21.29. The data intuitively reflects that older people living alone have poorer levels of mental health.

**Table 2:** Descriptive statistics of the variables.

Variables	Total	Living alone	Living with others
Depression	21.5383 (6.00221)	22.82626 (6.42434)	21.29176 (5.88658)
Living alone	0.16066 (0.36725)	1	0
Fruits	2.62691 (1.08278)	2.42727 (1.08499)	2.66512 (1.07826)
Vegetables	3.63032 (0.66832)	3.54242 (0.73873)	3.64714 (0.65271)
Nuts	2.36936 (1.39313)	2.17172 (1.34112)	2.40719 (1.39981)
Gender	0.48572 (0.49984)	0.37374 (0.48404)	0.50715 (0.5)
Age	80.13957 (10.67014)	81.83535 (9.31475)	79.81497 (10.88112)
Marital status	0.5344 (0.49886)	0.06768 (0.25132)	0.62374 (0.48449)
Years of schooling	4.60467 (4.57967)	3.52525 (4.21021)	4.81129 (4.61874)
Residence	0.3864 (0.48696)	0.41515 (0.493)	0.38090 (0.48565)
Perceived income adequacy	0.88721 (0.31636)	0.87576 (0.33002)	0.8894 (0.31366)
Visits	2.91091 (1.79722)	2.99192 (1.99695)	2.8954 (1.75619)
Self-rated health	3.5224 (0.89738)	3.50909 (0.90018)	3.52494 (0.89691)
Drink	0.16439 (0.37066)	0.1596 (0.36642)	0.16531 (0.3715)
Iadl	3.41237 (5.02959)	2.8899 (4.12531)	3.51237 (5.17906)
N	6,162	990	5,172

Data shown are means and standard deviations.

In terms of healthy diet and other covariates, older people living alone had lower consumption of fruits, vegetables, and nuts than older people who did not live alone. In addition, regarding the consumption of the three foods, vegetables were consumed the most. In contrast, nuts were consumed the least, possibly related to the deterioration of dental function in older people. In terms of covariates, older people living alone had a high mean age, a higher proportion of females, a lower proportion of marriage, fewer years of schooling, poorer economic conditions, and poorer self-rated health; however, older people living alone had lower IADL values, a lower proportion of alcohol consumption, and higher index of children’s visits.

### 4.2 Overall sample regression

We used two multiple regression equations in the overall sample regression to examine the effect of living alone on depressive symptoms, and the results are presented in **Table 3**. In the first model, we used the living alone variable and covariates. In model 2, we used the living alone variable, covariates, and healthy diet variables.

**Table 3:** Regression results of the effect of living alone on depression.

Variable	Model 1	Model 2
Alone	1.20945*** (0.20579)	1.0223*** (0.20375)
Gender	-0.42682*** (0.15199)	-0.57948*** (0.15088)
Age	-0.01098 (0.0091)	-0.0126 (0.00899)
Marital status	-0.357** (0.17561)	-0.37535** (0.17339)
Years of schooling	-0.08984*** (0.01707)	-0.04276** (0.01762)
Residence	0.06918 (0.1441)	-0.04933 (0.14317)
Perceived income adequacy	-2.13309*** (0.21929)	-1.98125*** (0.21705)
Visits	-0.1061*** (0.03999)	-0.12102*** (0.03953)
Self-rated health	-2.54942*** (0.07827)	-2.42528*** (0.07815)
Drink	-0.3478* (0.19156)	-0.33852* (0.18947)
Iadl	0.14042*** (0.01786)	0.12763*** (0.01772)
Fruits		-0.24613*** (0.06817)
Vegetables		-0.93559*** (0.10354)
Nuts		-0.27826*** (0.05079)
N	6,162	6,162
Adjusted R-squared	0.23	0.2494

*, P<0.1; **, P<0.05; ***, P<0.01.

In Model 1, the variance inflation factors for all variables are below 3, ranging from a minimum of 1.07 to a maximum of 2.09; in Model 2, the variance inflation factors for all variables are below 3, with a minimum of 1.07 and a maximum of 2.1, which is much lower than 10, showing that multicollinearity was not a problem in our regression models (81).

The regression results of Model 1 show that living alone positively affects depression values, increasing to 1.21 in depression scores, and the coefficient is significant. The variables of gender, marital status, children’s visits, self-rated health, years of schooling, and perceived income adequacy were all negatively associated with depression scores. The IADL variable had a positive effect on depression. The above regression coefficients are consistent with the results of most studies.

The results of Model 2 show that the coefficient of the effect of living alone on depression becomes smaller at 1.02 with the introduction of the healthy diet variable, and the coefficients of the covariates change slightly compared to Model 1. All three variables of healthy diet had a negative effect on depression.

The results of Model 1 and Model 2 confirm the H1 hypothesis that living alone leads to a significant increase in depression scores.

### 4.3 Mediation analysis

**Figure 2:**
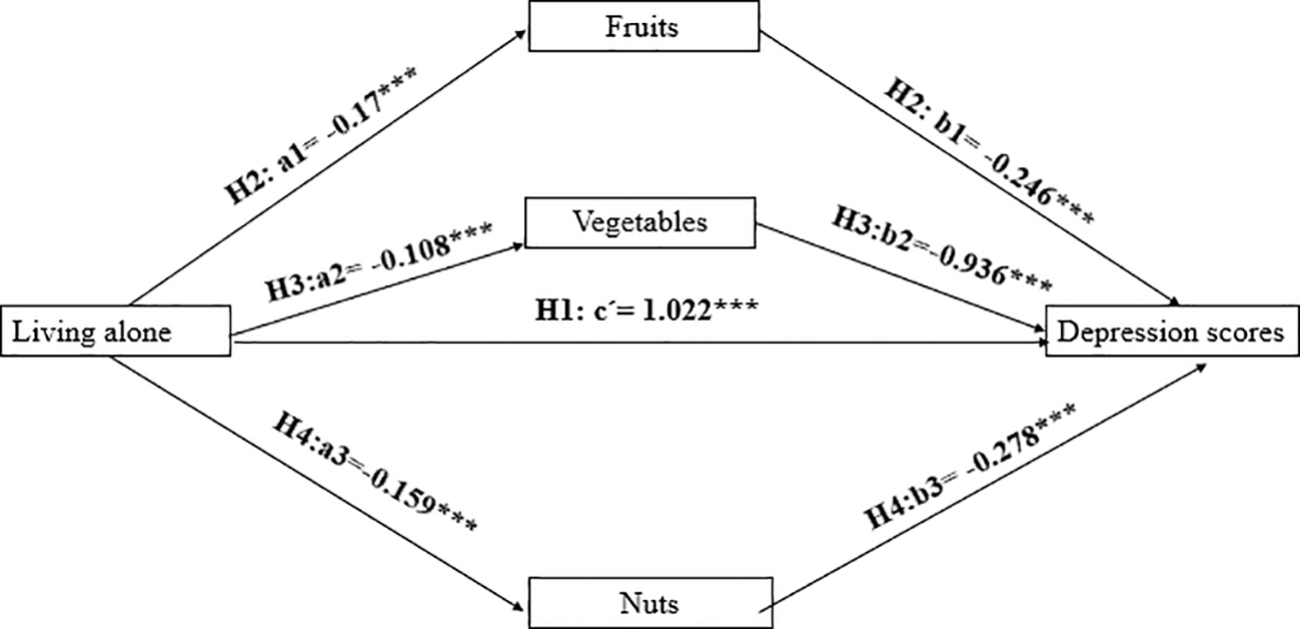
Path analysis. *, P<0.1; **, P<0.05; ***, P<0.01.

As hypothesized in the previous section, there are three pathways for the indirect effect of living alone on depression scores. We conducted path analyses using the Lavaan package in R software. The covariates are consistent with the regression equations described above. The path coefficients are shown in **Figure 2** below, where the paths for the effect of fruit consumption on depression values are denoted by H2a1 and H2b1, with living alone negatively correlated with fruit consumption, β= -0.17, P(>|z|)=0.000, and fruit consumption is negatively correlated with depression scores, β= -0.246, P(>|z|)=0.000. The paths for the effect of vegetable consumption on depression values are denoted by H3a2 and H3b2, where living alone is negatively associated with vegetable consumption, β=-0.108, P(>|z|)=0.000, and vegetable consumption is negatively associated with depression scores, β= -0.936, P(>|z|)=0.000. The paths for the effect of nuts consumption on depression values are represented by H4a3 and H4b3, where living alone is negatively associated with nuts consumption, β= -0.159, P(> |z|)=0.002, and nuts consumption is negatively associated with depression scores, β= -0.278, P(>|z|)=0.000.

The coefficients of the above path analyses are all significant, but statistical tests are needed to confirm the existence of mediating effects. And some scholars believe that bootstrapping was a good test. It has been argued that bootstrapping is a reasonable method for obtaining confidence intervals for the indirect effect under most conditions (82). The mediation effect test is generally a test of the significance of the product of two coefficients a*b (83).

Therefore, we use 1,000 times of bootstrapping to test the mediation effect and obtain a 95% confidence interval (CI). The results are shown in **Table 4** below. None of the upper and lower bounds of the confidence intervals of the bootstrapped a*b coefficients contain 0. Therefore, the mediating effect holds, and all three hypotheses, H2, H3, and H4, are valid.

**Table 4:** The mediation effect test.

Path	a*b	P(>|z|)	Boot Ci.lower	Boot Ci.upper
a1*b1: Living alone→ Fruits→ Depression scores	0.042	0.008	0.017	0.075
a2*b2: Living alone→ Vegetables→ Depression scores	0.101	0.000	0.05	0.163
a3*b3: Living alone→ Nuts→ Depression scores	0.044	0.008	0.016	0.08
Total effect	1.209	0.000	0.818	1.599

## 5 Discussion and policy recommendations

### 5.1 Main findings

To the best of our knowledge, this is one of the few studies focusing on the relationship between living alone, healthy diet, and mental health based on a sample of older adults in China. This paper expands the feasible perspective of mental health interventions for older adults living alone.

First, our study found that living alone can have a detrimental effect on the mental health of older adults. This is consistent with many existing studies; for example, a study in northern China found that the regression coefficient of living alone on depressive symptoms in older adults was 2.695 (84). Another regression analysis found a significant association between living alone and depressive symptoms in older adults, β= 0.842 (85). As the description of the data in the previous section shows, very few older adults living alone have spouses and, therefore, lack spousal support; in addition, older adults living alone have a lower level of education. As a result, living alone often leads to a deterioration in the mental health of older persons.

Second, healthy eating played an important mediating role between living alone and older adults’ mental health. Healthy eating affects the mental health of older adults through three channels, namely, fruits, vegetables, and nuts, respectively, and the indirect effects of these three channels accounted for 15.47% of the total impact of living alone on depression scores of older adults. Among them, vegetable intake had the most significant effect on depression scores, accounting for 8.35% of the total impact, followed by nuts and fruits. The reason may be that vegetables are an important feature of the traditional Chinese diet (86). Our findings are consistent with those of many studies, such as one study found that the higher the intake of vegetables and fruits, the lower the risk of depressive symptoms (87). This finding is important because the intake of vegetables, fruits, and nuts, among others, is related to ease of purchase and the financial income of older people. And in urban China, most families can buy fresh vegetables within walking distance (88). In rural China, however, the situation becomes different. Traditionally, rural households consume mainly home-grown food (89). Nowadays, although rural residents have also begun to go out to buy food, the retail system in rural areas is not sound, and supermarket chains and rural e-commerce are still underdeveloped, unable to meet the growing material needs of the rural population (90). Therefore, older adults living alone in rural areas face more difficulty consuming healthy food. In practice, we find that rural Chinese residents consume less vegetables and fruits than urban residents (91).

### 5.2 Policy recommendations

Based on our study, we make the following recommendations. Firstly, as China’s aging process deepens, compounded by a declining fertility rate, more and more older people will live alone in China. This is a large and vulnerable group, and high priority should be given to improving their mental health to achieve healthy aging. Second, some older adults living alone have a poorer financial situation, especially in rural areas. The government should care about them and provide livelihood protection to low-income older adults so that they can afford to buy healthy food.

Finally, given the important mediating role of healthy eating in the relationship between living alone and mental health, attention should be given to facilitating the purchase of healthy food for older persons at the community level. For example, food service vouchers can be provided to older adults living alone, and commercial organizations can be introduced to provide older adults living alone with services such as purchasing vegetables, fruits, and nuts on their behalf and cooking for them. In addition, a study in China, for example, found that community canteen services can improve the general mental health of older adults (92). Therefore, we propose to set up canteens in communities with many older adults living alone so that they can eat more healthily, thereby improving their mental health.

### 5.3 Limitations

Our study also has shortcomings; first, we derive a statistical relationship between the variables. However it is difficult to conclude a causal relationship between the variables due to the use of cross-sectional data; therefore, more in-depth analysis using panel data should be conducted in the future. Secondly, the questionnaire did not inquire in more detail about how and where the older adults living alone purchased fruits and vegetables, so we could not infer the objective reasons affecting the consumption of fruits and vegetables by older adults living alone. Third, the research questionnaire did not include more detailed information on the consumption of fruits, vegetables, nuts, etc., but only on the frequency of consumption; similarly, the assessment of alcohol intake does not indicate the frequency and amount of intake and future studies should use more detailed data for analysis. At the same time, older adults living alone may also have problems with their diet, such as consuming stale leftovers. Therefore, more in-depth research should be conducted on the nutritional elements that affect the mental health of older adults (93).

## 6 Conclusion

This study identifies mechanisms by which healthy eating mediates the relationship between living alone and mental health. It was found that older people living alone are a vulnerable group with poorer mental health and a lower intake of healthy foods such as fruits, vegetables, and nuts, which can have an impact on their mental health. The findings suggest that healthy eating is a feasible intervention to improve the mental health of older adults living alone and that the Government can provide low-income older people living alone with livelihood protection so that they can afford to buy healthy food; at the same time, the Government should take diversified initiatives at the community level to enhance the healthy diet of older persons living alone.

## Data Availability

Publicly available datasets were analyzed in this study. This data can be found here: https://opendata.pku.edu.cn/dataset.xhtml?persistentId=doi:10.18170/DVN/WBO7LK.
